# Land cover change explains the increasing discharge of the Paraná River

**DOI:** 10.1007/s10113-018-1321-y

**Published:** 2018-04-09

**Authors:** Eunjee Lee, Angela Livino, Shin-Chan Han, Ke Zhang, John Briscoe, Jerson Kelman, Paul Moorcroft

**Affiliations:** 1 Sustainability Science Program, Kennedy School of Government, Harvard University, Cambridge, MA USA; 2 Present Address: Goddard Earth Sciences Technology and Research, and Global Modeling and Assimilation Office, NASA Goddard Space Flight Center, Greenbelt, MD USA; 3 Present Address: Energy Research Office, Empresa de Pesquisa Energética (EPE), Rio de Janeiro, 20.090-003 Brazil; 4 School of Engineering, The University of Newcastle, Callaghan, NSW Australia; 5 State Key Laboratory of Hydrology-Water Resources and Hydraulic Engineering, and College of Hydrology and Water Resources, Hohai University, Nanjing, 210098 China; 6 Federal University of Rio de Janeiro, COPPE-UFRJ, Civil Engineering Program, Rio de Janeiro, RJ Brazil; 7 Department of Organismic and Evolutionary Biology, Harvard University, Cambridge, MA USA

**Keywords:** Land cover change, River discharge, Paraná River basin, Shift of the peak discharge timing, Hydroelectricity generation in Brazil

## Abstract

Over the past 40 years, the discharge in South America’s Paraná River basin has increased despite no evidence of significant rainfall increases in the basin. In this analysis, we show that the observed multi-decadal increase in discharge can be explained by concomitant changes in land cover that have occurred within the basin during this period. Our analysis also indicates that the peak discharge timing may have shifted concurrently from January/February in the 1970s to March in more recent decades. While land-use effect dominantly alters the long-term temporal dynamics of the river discharge over multi-decades, the change in the seasonality of the discharge can be attributable to the combined effect of the land-use and climate variability. This study suggests that the mean annual discharge is likely to change in the other South American River basins where land transformation is currently taking place, and the shift of the month of peak discharge needs to be taken into consideration to forecast the hydropower generation under changing climate and land conversion.

**Electronic supplementary material:**

The online version of this article (10.1007/s10113-018-1321-y) contains supplementary material, which is available to authorized users.

## Introduction

Hosting multiple hydropower dams that generate a significant portion of electricity to meet the regional demand in southern Brazil, the Paraná River basin is geographically and economically important. The famous Itaipu hydropower plant, built in the lower reaches of the upper Paraná River basin at the Brazil-Paraguay border, has the installed capacity of 14,000 MW (MW), yielding the electricity that supplies 15% of Brazil’s total energy consumption (https://www.itaipu.gov.br/en).Considering the country’s heavy dependency on hydropower (up to 80%, U.S. Energy Information Administration 2014), the variability of the flow in the Paraná River basin is undoubtedly critical for the region’s sustainable energy supply.

Over the past several decades, the mean discharge of the Paraná River has increased notably (Tucci and Clarke 1998; Dai et al. 2009; Carvalho et al. 2011). Having the 1970s as the baseline period, the reconstructed natural flow inferred from the measurements from the gauge station at the Itaipu dam (25.43 S, 54.59 W) affirms the trend: + 11.1% in the 1980s, + 18.0% in the 1990s, and + 6.3% in the 2000s (data from ANA, the Brazilian National Water Agency, http://www2.ana.gov.br/). However, there is no evidence of any significant increase in rainfall over this period. The baseline precipitation of the 1970s, from the combination of reanalysis and observation-based datasets (Sheffield et al. 2006), indicates that mean annual rainfall decreased by 1.5% in the 1980s (or by 5.0% excluding the rainfall of the 1983 flood event as an outlier), increased by 4.2% in the 1990s, and then declined slightly by 1.0% in the 2000s.

On the other hand, the basin has undergone historically extensive land transformation. In the state of Paraná, forest cover decreased from 23.9% in 1965 to 5.2% in 1990, being replaced by annual crops since the 1970s (Tucci and Clarke 1998). The relationship between land cover and river discharge has been reported previously, including the historical debates (review in Andréassian 2004) and the early work by Bosch and Hewlett (1982). In particular, the alteration of river flow associated with deforestation in tropical basins was observed for a large number of watersheds (review in Farley et al. 2005; Oudin et al. 2008). For instance, Coe et al. (2009) suggested that the degree of vegetation removal and the deforestation rate of particular watersheds affect the discharge of the Amazon River basin. The land cover change was also shown to alter the discharge flows in its tributaries such as the Toscantins River (Costa et al. 2003), the Ji-Paraná River (Rodriguez et al. 2010; Rodriguez and Tomasella 2016), and the Xingu River (Dias et al. 2015; Panday et al. 2015). Despite the large body of literature that suggested the altered river flows of the individual drainage basins in South America, the historical change in discharge of the Paraná River has received little attention.

In this paper, we examined the mechanistic linkages between climate variability, land-use, and resulting river discharge in the Paraná River basin using a terrestrial biosphere model, the Ecosystem Demography version 2 (ED2) (Moorcroft et al. 2001; Albani et al. 2006; Medvigy et al. 2009). The model results were evaluated against the natural flows at Itaipu and the regional total water storage (TWS) change from the NASA’s Gravity Recovery and Climate Experiment (GRACE) satellite observation. We then demonstrated that land transformation indeed accounts for the decadal increases in discharge of the upper Paraná River basin that have occurred in the past 40 years, despite little or no change in the basin’s rainfall. The potential shift of the seasonality of the river discharge, which is another important indicator to measure the hydrologic change (Döll and Schmied 2012), is also explored.

## Methods

### ED2 model

The ED2 model calculates the water, carbon, and energy dynamics of the land surface. One of ED2’s distinguishing features is its ability to describe, in a physically consistent manner, the coupled water, carbon, and energy dynamics of heterogeneous landscapes. This ability to incorporate the land-use change and resulting heterogeneous patterns of land cover, and its impacts on the hydrological processes, ecology, and energy balance means that the model is ideally suited for investigating the combined impacts of rainfall trends and land-use change within the upper Paraná River basin.

The heterogeneous landscape of the region was represented by the mixtures of primary forest, secondary forest, and agricultural area. Four plant functional types (PFTs) were used: (1) early successional trees (fast growing, low density, water demanding), (2) mid-successional trees, (3) late successional trees (slow growing, high density, shade tolerant), and (4) C4 type grass. While a grid-cell shared a homogeneous meteorological forcing, evaporation fluxes were computed from the sub-divided, multi-layer canopy structure by size and age within a grid-cell. The stomatal conductance that regulates the plant’s transpiration was calculated by the water vapor deficit functions (Leuning 1995). A multi-layer soil model (Walko et al. 2000) was used to simulate surface runoff, soil moisture, and drainage. With the land-use disturbance rate updated annually, the model simulated the hydrologic changes associated with sub-grid heterogeneity of vegetation dynamics in the model time steps that span from seconds to 5 min (e.g., energy and water cycles) up to 15 min (e.g., photosynthesis and soil respiration). For further description of the ED2 model, please see Medvigy et al. (2009), and also Knox et al. (2015) and Swann et al. (2015) that used the ED2 model’s couple version to a regional climate model to investigate the past, current, and future land-use impact on hydroclimate of the surrounding regions in South America.

### Study domain and experimental design

**Fig. 1:**
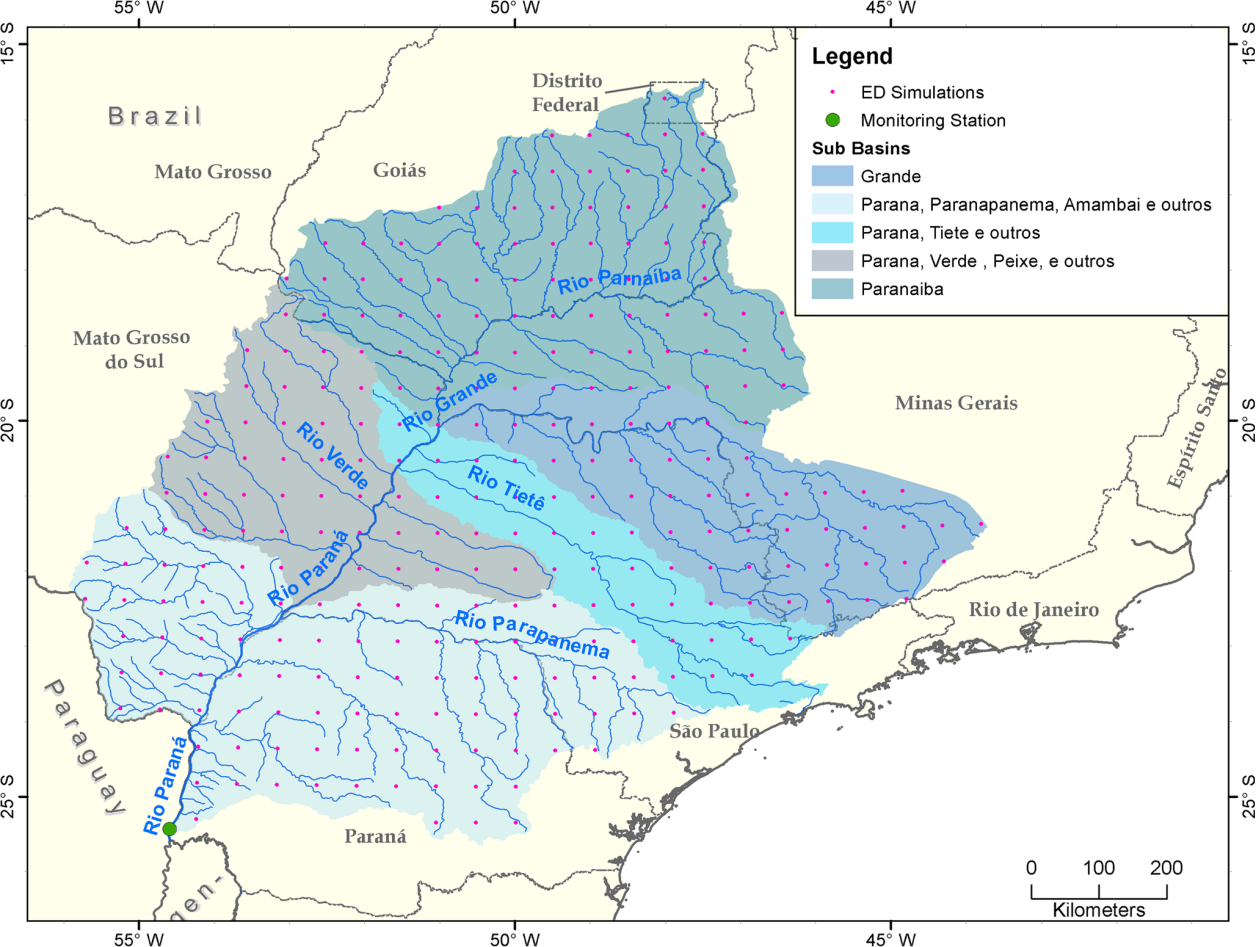
Map of the upper Paraná River basin. The discharge monitoring station is located at the Itaipu Dam (25.43 S, 54.59 W, green circle). Pink dots indicate the centers of grid-cells used for ED2 simulations

Model simulations were conducted for a region encompassing the five major sub-basins of the upper Paraná River: the Grande, Paranapanema, Tiete, Verde, and Paranaiba River basins (a total drainage area of approximately 787,000 km^2^) that feed the river discharge measured at the Itaipu Dam (Fig. 1). Note that the regional boundary of the basin of this study does not include the nearby Pantanal, the large-scale wetland area that consists of marsh.

**Fig. 2:**
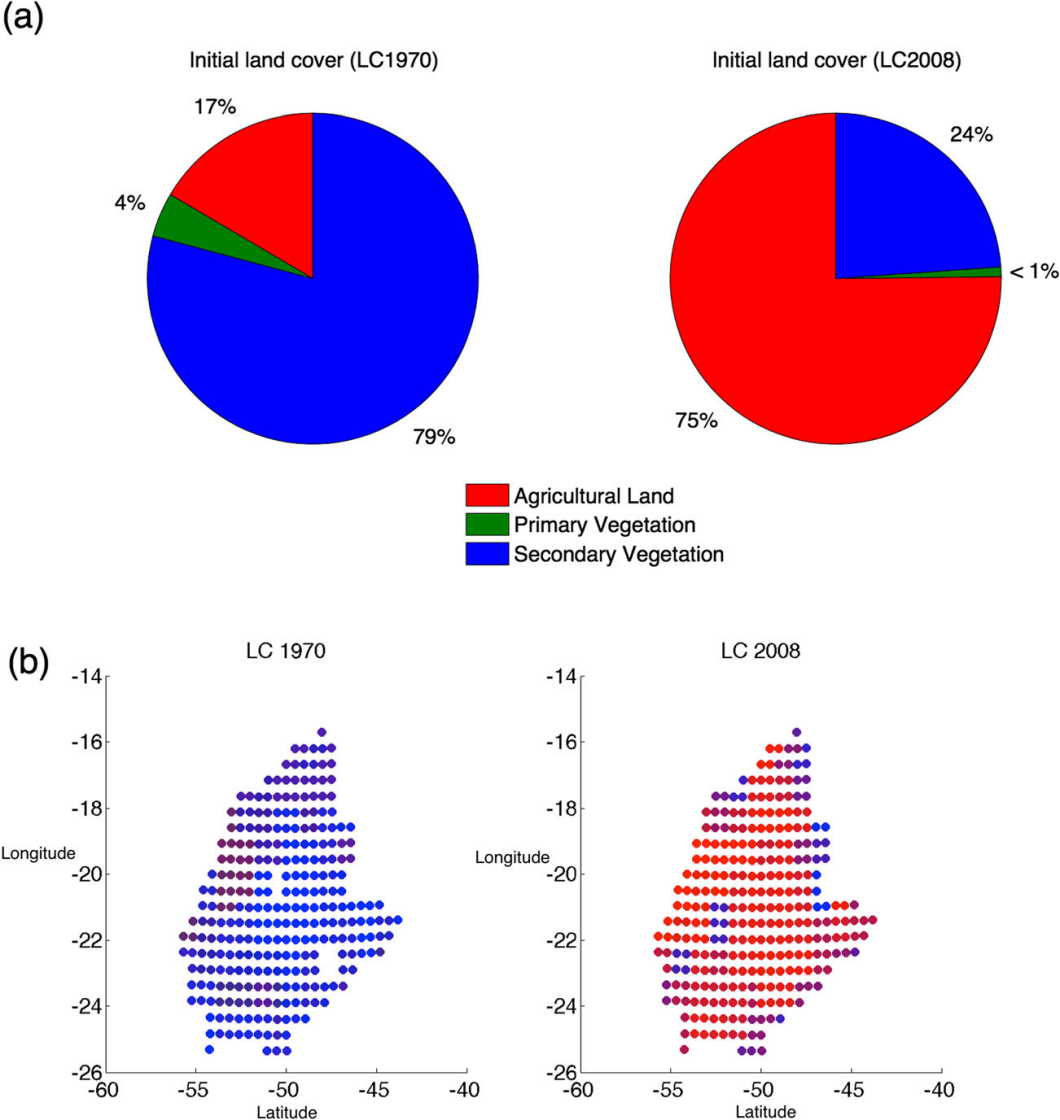
Initial land cover maps of LC1970 (Left) and LC2008 (Right): **a** Basin-wide fractions of agricultural land (red), primary vegetation (green), and secondary vegetation (blue), and **b** spatial patterns of the vegetation types. Primary vegetation means that its last disturbance is a natural event such as fire. Secondary vegetation means that its last disturbance is anthropogenic. Definitions of the disturbance type are as described in Albani et al. (2006)

Following the approach of Hurtt et al. (2006) and Albani et al. (2006), two representative ecosystem states, corresponding to the region’s land cover in 1970 and contemporary land cover patterns in 2008 (hereafter 1970LC and 2008LC, respectively), were developed by forcing the model with the historical land-use transition dataset of Hurtt et al. (2006). This dataset specifies the historical patterns of the land-use transitions between three land-use states: agricultural land, primary vegetation, and secondary vegetation. In 1970LC, the Paraná region consisted of 17% of agricultural land, 4% of primary forest, and 79% of secondary forest, while in 2008LC, the region was comprised of 75% of agricultural land, less than 1% of primary forest, and 24% of secondary forest (Fig. 2).

Five simulations (Table 1) were conducted at a spatial resolution 0.5**°** × 0.5**°** (approximately 50 km × 50 km) for 280 grid-cells spanning the upper Paraná River basin that contributes to the discharge at the Itaipu dam (green dot in Fig. 1). The pink dots in Fig. 1 correspond to the centroids of the grid-cells. The atmospheric CO_2_ concentration was kept constant at 378 ppm over the simulation period.

**Table 1:** Five simulations conducted in this study

Name	Initial land cover	Meteorological forcing
LCC-fullClim	Time series of annual land-use transitions applied for 1970–2007	1970–2008 (Sheffield et al. 2006)
1970LC-fullClim	Land cover state corresponding to the land-use pattern of the year 1970	1970–2008 (Sheffield et al. 2006)
2008LC-fullClim	Land cover state corresponding to the land-use pattern of the year 2008	1970–2008 (Sheffield et al. 2006)
LCC-70sClim	Time series of annual land-use transitions applied for 1970–2007	Cyclically repeated the 1970s climate
LCC-00sClim	Time series of annual land-use transitions applied for 1970–2007	Cyclically repeated the 2000s climate

In the initial simulation (LCC-fullClim), the model was forced with both the observed 1970–2008 time series of meteorological forcing specified from the dataset by Sheffield et al. (2006) and the abovementioned time series of land-use transitions that occurred during this period. The contributions of changes in climate forcing and land-use change to the pattern and magnitude of discharge at Itaipu were estimated by conducting two additional pairs of simulations. In the first pair (1970LC-fullClim and 2008LC-fullClim), the model was forced with the observed time series of meteorological forcing for the period of 1970 to 2008 specified from the dataset by Sheffield et al. (2006) but with the land cover states corresponding to either the land-use pattern of the year 1970 (1970LC) or present-day land-use patterns (2008LC). In the second pair of simulations (LCC-70sClim and LCC-00sClim), the model was forced with the Hurtt et al. (2006) time series of land-use transitions that occurred during the period 1970–2007, with either climate specified for the 1970s (LCC-70sClim) or for the 2000s (LCC-00sClim). The 2008 climatology was applied for the 10th year of each decade. In LCC-70sClim and LCC-00sClim simulations, land conversion rates were applied to introduce disturbances originating in human activity specified from the Global Land Use dataset, for 1970–1999 from Hurtt et al. (2006). We applied the rate of year 1999 onward until 2008 because of little change in total agricultural area for 2000–2008 within the basin. The LCC-70sClim was forced by the climatological condition of the 1970s, and the LCC-00sClim by the climatology of the 2000s. All simulations generated 39-year multi-decadal monthly time series on the predicted changes in the water cycle and its major components (i.e., patterns of water discharge, storage, and evapotranspiration) via the coupled water, carbon, and energy dynamics across the basin.

### Datasets and model evaluation metrics

The spatially integrated monthly discharge fluxes predicted by this scenario were then evaluated against the estimated natural flow at the Itaipu dam gauge station (25.43 S and 54.59 W). The natural flow was reconstructed from the daily discharge measurements at the Itaipu Dam, by taking into account the temporal gains and losses arising from reservoir operation and water withdrawals at the upstream of the gauge station (data available from the ANA (Brazilian National Water Agency) website (http://www2.ana.gov.br/). In addition, the model’s predictions of the temporal dynamics of total water storage (TWS) within the upper Paraná River basin were evaluated against the GRACE satellite measurements of this quantity (Rodell et al. 2004; Güntner 2008; Syed et al. 2008; Han et al. 2009). Because GRACE measures only relative differences in TWS, the respective mean values of TWS from model simulations were removed, and the anomalies were compared against the change in GRACE-estimated TWS over the period of 2002–2008. For large river basins such as the Amazon, the speed of the river flow must be factored into TWS storage calculations because the amount of laterally transported water within the basin is significant (Han et al. 2010). However, the relatively small size of the upper Paraná River basin and the monthly time scale of the analysis meant that river routing effects can be neglected, and thus the model’s predictions of TWS can be simply calculated based on the differences in the aggregate fluxes of water (i.e., precipitation minus evapotranspiration and runoff) within the river basin.

**Fig. 3:**
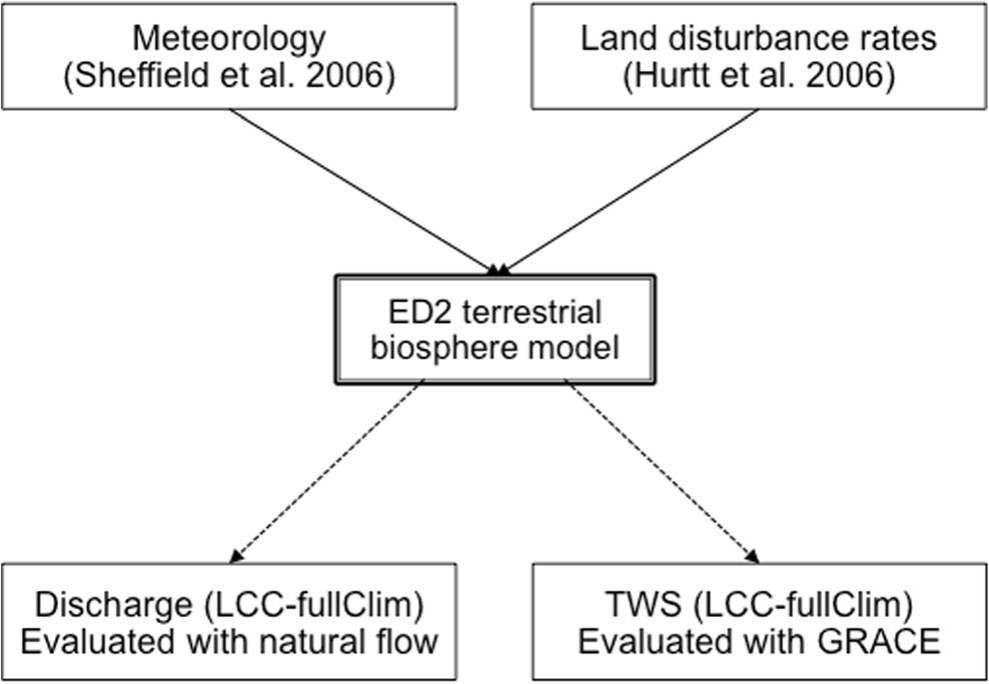
A schematic figure that demonstrates the ED2 model inputs (meteorology and land cover) and selected output variables (aggregated discharge and total water storage (TWS)), and measurement datasets (natural flow at Itaipu and GRACE satellite observation) used for the validation of the LCC-fullClim simulation

We applied the Mann-Kendall test with a confidence level of 95% for the trend analyses of the discharge at Itaipu Dam and the precipitation over the upper Paraná River basin. The goodness-of-fit of the ED2 model to GRACE was assessed with the Nash-Sutcliff Efficiency (NSE) value (Nash and Sutcliffe 1970), a popular measure of the goodness-of-fit between the model predictions and the measurements. A summary of the validation methodology is shown in Fig. 3.

## Results

### Model mean annual discharge validation

**Fig. 4:**
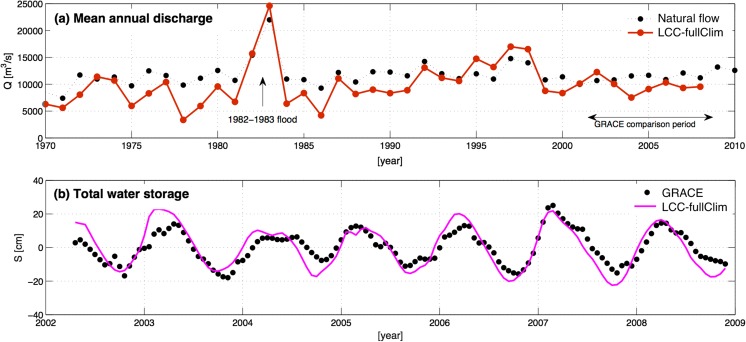
**a** Mean annual discharge flows of LCC-fullCLim case (solid red line) and the natural flow (dotted line with black circles) for the 1970–2008 time period, and **b** Total water storage (TWS) anomalies derived from the same case (LCC-fullClim, solid pink line), compared with the TWS anomalies derived from GRACE satellites measurement (circled black dots), from April 2002 (beginning of the GRACE observations) to December 2008 (the end of the climate forcing dataset)

Figure 4a shows the predictions of mean annual discharge from the LCC-fullClim simulation, the most realistic of the simulations that includes the impacts of both historical changes in climate forcing and land cover change. Overall, the ED2 model predicts well the pattern and magnitude of inter-annual variability in the observed discharges in the natural flow, including the large El Ninõ-linked discharge event in 1982–1983 that was one of the worst recorded floods in the Paraná River basin (Camilloni and Barros 2000). The inter-annual variability of the predicted discharge (red line) shows reasonable agreement with the natural flow at Itaipu (black dashed line); however, the model tends to be negatively biased (mean error of − 1703 m^3^/s) for the 39-year period.

The corresponding predictions of Total Water Storage (TWS) within the basin are shown in Fig. 4b. As can be seen in the figure, the model’s predictions (purple line) agree with GRACE observations of TWS anomaly (black dots) in timing and amplitude of seasonal peaks and troughs from 2002 to 2008. The Nash-Sutcliff goodness-of-fit value, a measure of degree of model’s agreement with observation, between the LCC-fullClim and GRACE, is 0.6, indicating plausible matches between the ED modeled flow and the GRACE observation. The model reproduces well the observed inter-annual pattern of the seasonal change in TWS, such as larger peak-trough changes in 2003 and 2007 compared to those in 2004–2006, and the prolonged seasonal increase in storage that occurred in 2004.

### Land-use impact on mean annual discharge increase over the last decades

The natural flow at Itaipu (green dot in Fig. 1) showed that in the 1970s baseline period, the annual discharge was 10,474 m^3^/s, but increased to 12,677 m^3^/s in the 1980s (a + 21.1% increase, or excluding the anomalously large 1983 flood year, a + 11.1% increase), to 12,138 m^3^/s the 1990s, and to 11,135 m^3^/s in the 2000s—increases of 18.0 and a 6.3%, respectively (Table A1). For the 39-year mean annual discharge over the period of 1970–2008, the increasing trend is not statistically significant because of the leveled-off discharge after 1998; however, if we consider only the non-PDO period (1970–1998), the Mann-Kendall test shows that the discharge increase remains statistically significant with a confidence level of 95%. According to Dai et al. (2009), this suppression may be the consequence of a large-scale climatic phenomenon, the Pacific Decadal Oscillation (PDO). If we consider all the available years (1931–2010) of the discharge measurement at Itaipu, the increasing trend of the annual discharge is statistically significant with a confidence level of 95%. This reaffirms the results by Dai et al. (2009) and Carvalho et al. (2011); nevertheless, the domains of their studies are slightly different from this work.

**Fig. 5:**
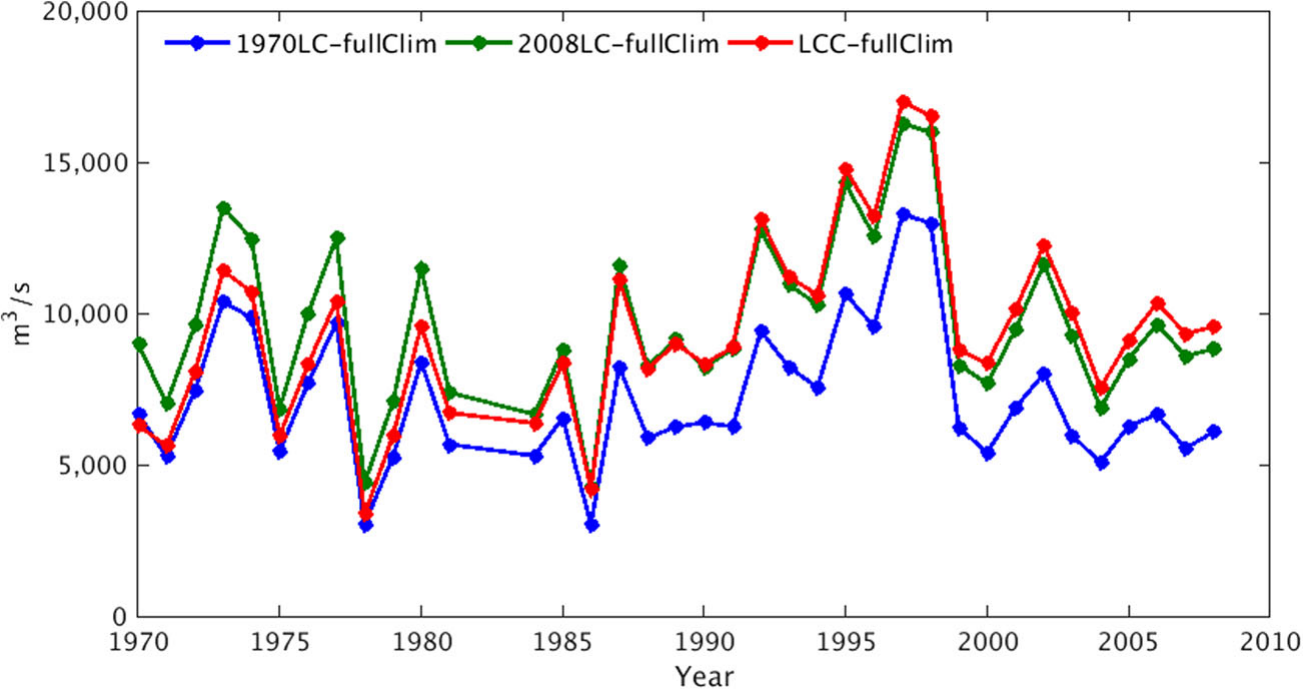
ED2 model discharges from the LCC-fullClim case (red curve) compared with the simulations that kept 1970 land cover status (1970LC-fullClim, blue curve) and the 2008 land cover status (2008LC-fullClim, green curve). The outliner values of 1982 and 1983 during the flood were excluded in this figure

Figure 5 demonstrates the patterns of mean annual discharge at Itaipu predicted by the 1970LC-fullClim simulation in which there is no land cover change after 1970, and the LCC-fullClim simulation that incorporates the effects of historical changes in both climate forcing and land cover (blue and red lines, respectively, in Fig. 5). If we apply only the climatic variability and do not account for the effect of historical land-use that took place after year 1970, the discharge from the 1970LC-fullClim (blue curve) does not show a statistically significant increase for the period of 1970–1998. By accounting for the historical land cover change (i.e., LCC-fullClim), the positive trend becomes statistically significant with 95% confidence level. Therefore, it is not likely that the climatic variability alone does cause the increasing trend of the annual discharge during the last four decades in the Paraná River basin. On the other hand, the discharge from the 2008LC-fullClim was higher in the early part of the simulations (e.g., the 1970s) and remains relatively flat compared to LCC-fullClim (red). This affirms our hypothesis that the historical land cover change is a primary driver to increase the annual discharge of the Paraná River basin for the last four decades.

The predicted rates of discharge after the mid-eighties are considerably smaller in the absence of land cover change, 73.9% of discharge assuming no land cover change (9047 m^3^/s from the 1970LC-fullClim and 12,237 m^3^/s from the LCC-fullClim) in the 1990s, and 64.5% discharge assuming no land cover change (6210 m^3^/s from the 1970LC-fullClim and 9627 m^3^/s from the LCC-fullClim) in the 2000s, which suggests that land cover change has contributed to an increased rate of discharge of the basin and that climate variability alone cannot explain the observed increases in rates of discharge that have occurred since the mid-eighties (Fig. 5). On the other hand, assuming the absence of the land-use disturbance, the climate variability over the historical period causes discharge to slightly decrease in the 1980s (− 1.0%), increase in the 1990s (+ 27.9%), and decrease again in the 2000s (− 12.2%) compared to the rates of discharge during the 1970s (Table A2).

### Seasonality shift of mean monthly discharge

**Fig. 6:**
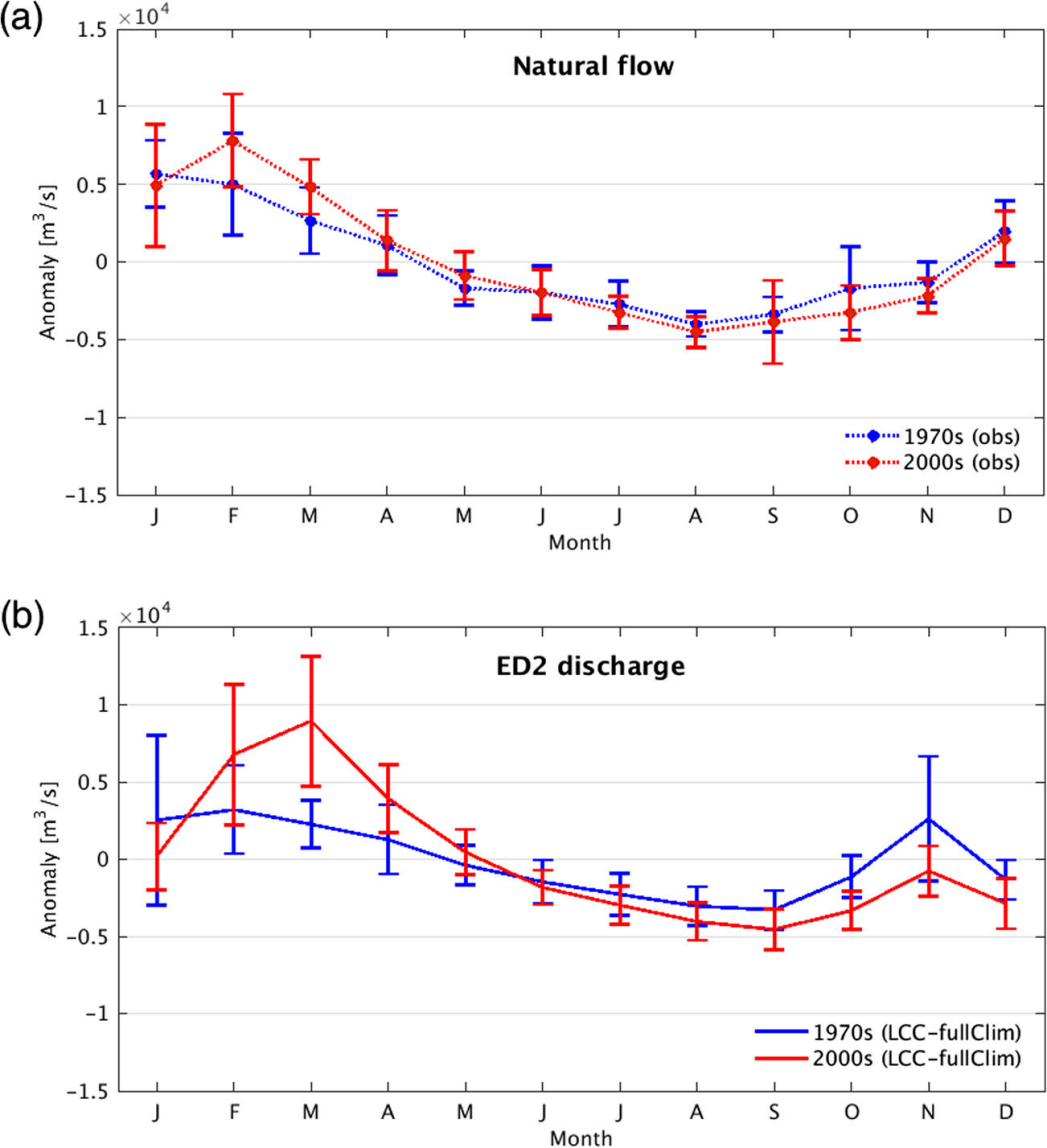
**a** Seasonal variability of the discharge anomaly of the natural flow reconstructed from the measured flow at Itaipu, and **b** from the LCC-fullClim. Long-term trends of decadal and inter-annual variability were removed by subtracting each 12-month running mean value. Error bars represent standard deviations

Alongside the increase in the historical mean annual discharge, we demonstrate that there may also have been increase shifts in the seasonal pattern of discharge. In Fig. 6a, the monthly discharge anomaly of the natural flow at Itaipu indicates a shift of the peak monthly flow from January in the 1970s (blue lines) to February in the 2000s (red lines). The shift was also observed in the 1980s and 1990s (Fig. A1). Likewise, the seasonality of the discharge anomaly of the ED2 model (Fig. 6b) indicates the shift of the month of the maximum discharge at Itaipu in the 1970s and 2000s predicted by the LCC-fullClim simulation. Consistent with the observed shifts towards later peak in flow seen in Fig. 6a, the month with the highest rate of discharge shifts from January /February in the 1970s (blue line) to March in the 2000s (red line). Moreover, the greater amounts in seasonal flows have been observed in March, April, May, and June. The increasing trends of the natural flow are statistically significant (Mann-Kendall test, *p* < 0.05%) across the 1970 to 2008 period in March, and also before the PDO in the April, May, and June (Fig. A2).

**Fig. 7:**
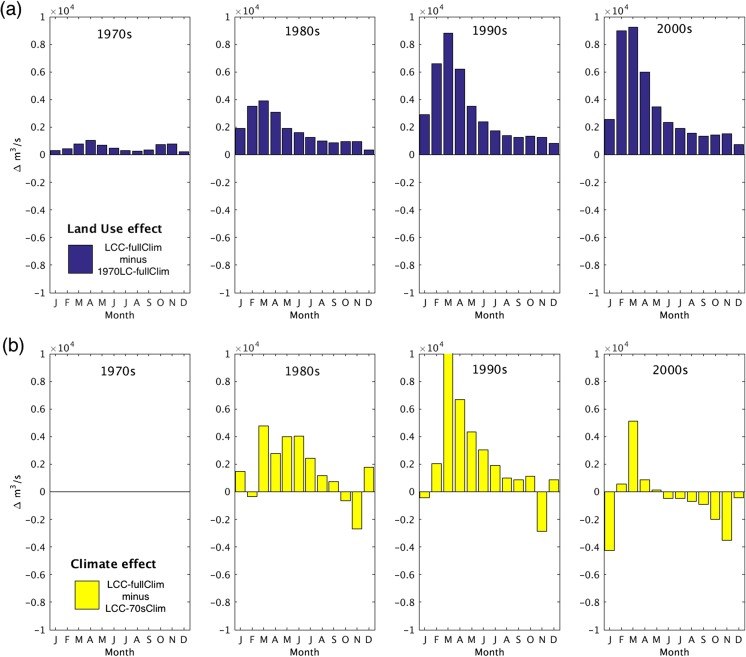
**a** Isolated land-use effect and **b** isolated climate variability effect to the decadal mean monthly discharge. The blue bars represent anomalies of each decadal mean discharge of LCC-fullClim compared to the 1970LC-fullClim, demonstrating a consistency in discharge increase by the land-use effect. The yellow bars show the anomalies compared to the LCC-70sClim

Given the same climate (fullClim), the comparison of the two model simulations (LCC-fullClim vs. 1970LC-fullClim) suggests that the land-use effect also causes increased amounts of monthly discharge in the basin throughout the 1980s, 1990s, and 2000s (Fig. 7a). By isolating the climate effect only (LCC-fullClim vs. LCC-70sClim), the consistent increase in the monthly discharge anomalies is not clearly shown (Fig. 7b), however means discharge increase in spring and summer (e.g., a larger amount of the monthly discharge shown in March), in particular during the 1980s and 1990s. The positive trend of the rainfall in the preceding months (February and March) are found to be statistically significant (time series of rainfall for each month shown in Fig. A3), which suggests the combined effect of land-use and climate variability may have triggered the shift of this temporal dynamics of the monthly peak discharge. Therefore, climate variability contributed to the discharge shift in seasonality and it was amplified by the land-use effect.

## Discussion

Our results indicate that the mean annual discharge increase recorded over the past few decades in the upper Paraná River basin is primarily attributable to the expansion of agricultural land as a result of conversion from forests (Fig. 5). The amount of annual discharge increases about 28% (1970LC-fullClim vs. LCC-fullClim) with the 4.4 times expansion of the agricultural land (from 17 to 79% in Fig. 2a). Our findings agree with previous studies showing that land conversion from forest to agriculture can increase the discharge: at a small watershed in Upper Xingu River basin, land conversion from forest to soybean cultivation increased mean daily discharge about four times, as well as the variability between the wet season and the dry season (Hayhoe et al. 2011), and in a comparison study of two catchments in eastern Amazonia, the pasture catchment had higher discharge than the forest catchment (de Moraes et al. 2006). Our findings are also consistent with the studies using dynamic global vegetation models, which showed that the land-use effects have had increased runoff of the twentieth century in eastern Brazil (Gerten et al. 2008) and the tropics (Piao et al. 2007).

The shift of the month of the maximum discharge (Fig. 6) corresponds to the positive increase in rainfall in earlier months (February and March, see Fig. A3). The intensification of the wet season precipitation and the peak river discharge over the past several decades was shown over the Amazon River basin (Gloor et al. 2015), as well as the altered rainfall seasonality, for example, the delayed onset of the wet season at Rondonia in eastern Amazon (Butt et al. 2011) and an increase in dry-season length over southern Amazonia (Fu et al. 2013). Our results suggest that the inclusion of both land conversion and the variability and trends in climate explain the observed increase in the monthly and seasonal discharge anomalies in the Parana River basin (Fig.6 and Fig. 7).

Associated with the land clearing from 1970s to 2000s in our simulations (Fig. 2), the basin-wide mean monthly ET is predicted to decrease by the range of 0.1 to 0.6 mm per day (Fig. A4). In the ED2 model, the root depth decreases from 2~3 m for trees to 1 m for agricultural land, thus limiting the available water supply from deep soil, regulating the size of open stomata, and reducing the transpiration flux (a component of ET). The reduction in ET, triggered by the changes in root depth, may lead to a greater amount of discharge. There are other changes that occur with land transformation, such as changes in Leaf Area Index (LAI), biomass, and PFTs that can also affect ET. Note that the effects of irrigation are not included in the ED2 simulations. Because the regional agriculture of the Paraná basin is mostly rainfed and irrigation is applied to the very limited part of the basin, our model discharge is directly comparable to the natural flow. However, for the regions where the irrigation plays a major role in growing crops, the plants’ water stress is greatly reduced and the change in ET with land conversion is not expected as much as our result is demonstrated. For such regions, the relationship between the ET change and the discharge variation, associated with the land cover change, have to be more carefully interpreted by considering the impacts of irrigation.

The reduced ET associated with land conversion may also contribute to the precipitation reductions that have occurred in the neighboring regions of southern and southeastern Amazonia (Doughty et al. 2012; Gloor et al. 2013). Studies using the coupled version of the ED2 model that represents the water and energy feedback processes (ED-BRAMS model) suggest, however, that historical land conversion of the northern South America is not necessarily related to the regional pattern of the precipitation anomaly (Knox et al. 2015). A recent coupled biosphere-atmosphere modeling study indicates that the feedback arising from future land cover change over Amazonia does not immediately trigger the precipitation change, but rather alters the status of the atmosphere more suitable for convection (Swann et al. 2015). Therefore, the ET reduction of the upper Paraná River may contribute to the continental-scale feedback to the atmosphere; however, the extent and magnitude of the precipitation feedback arising from the land transformation analyzed in this study is subject for future research.

From an applied perspective, these changes in discharge of this basin have important implications for the hydropower generation potential of the Paraná River basin. For example, assuming a business-as-usual deforestation scenario, the effect of increasing mean discharge on the enhanced hydroelectric energy generation was predicted to be about 10% for a hydroelectric dam on the Xingu River (Stickler et al. 2013). On the other hand, however, the altered seasonality of the discharge also affects hydropower generation and offsets the advantage of the increased mean annual discharge because in the wet season, once the water level reaches the maximum dam storage capacity, the excess water must be released to prevent from spilling over and cannot be used for electricity generation. If a delayed onset and shortening of the wet season (e.g., Fu et al. 2013) occurs, the number of days for stable operation of hydroelectricity generation reduces and a sustainable power generation declines.

## Conclusion

The ED2 model of this study is shown to reproduce reasonably well the observed discharge increase at Itaipu and the regional TWS derived by GRACE satellite measurement. This study affirms the significance of land-use change effects for estimation of the regional water budget of the South American River basins, and therefore contributes to the scientific community’s ongoing efforts to understand the impacts of land-use and climate change on the future variability of the South American hydrology. The terrestrial feedback of the water and energy cycles onto the atmosphere can alter the status of the atmosphere and potentially the rate and pattern of rainfall: both of which, in turn, can indirectly influence the aggregated values of regional discharge and water storage. Our result on the altered mean annual volume and the seasonality of discharge in the Paraná River basin, which has a history of an intensive land conversion over the past decades, suggests reevaluation of the stationary assumption on rainfall and discharge seasonality that has been applied in planning the hydropower plants in other river basins in South America. In the era of unprecedented large-scale land conversion and climate change, the complicated, and often paradoxical, response of the hydrologic cycle can be unraveled with the help of the terrestrial biosphere models that incorporate both land cover and climate change processes.

## Electronic supplementary material

ESM 1 (DOCX 313 kb)
