# Supplementary material for: Land cover change explains the increasing discharge of the Paraná River
Source: Reg Environ Change. 2018 Apr 9;18(6):1871–81. doi: 10.1007/s10113-018-1321-y (PMC6434983; doi:10.1007/s10113-018-1321-y)
Supplement: Supplementary file 1 — (DOCX 313 kb) [file 10113_2018_1321_MOESM1_ESM.docx]

**Appendix**

**Table A1**. Summary of mean annual natural flow measured at the Itaipu (data from ANA), and mean annual rainfall from Sheffield et al. (2006). The columns of relative change indicate the change relative to the mean value of the 1970s.

| Decade | Mean annual discharge (m^3^/s) | Relative change to the 1970s (%) | Mean annual rainfall (mm/year) | Relative change to the 1970s (%) |
| --- | --- | --- | --- | --- |
| 1970s | 10,474 | − | 1,502 | − |
| 1980s^*^ | 11,641 | +11.1 | 1,428 | −5.0 |
| 1990s | 12,358 | +18.0 | 1,565 | +4.2 |
| 2000s^+^ | 11,135 | +6.3 | 1,487 | −1.0 |

* 1983 (the flood event) was excluded as an outlier and thus 1980s is the 9-year average of (1980-1989).

+ The mean discharge of the 2000s is the 9-year average of (2000-2008).

**Table A2.** Predicted discharges of the four ED simulations. The first set of two simulations (1970LC-fullClim and 2008LC-fullClim) illustrates the discharge change due to the isolated effect of climate variability. The second set of simulations (LCC-70sClim and LCC-00sClim) highlights the isolated effect of land-cover change by both natural and anthropogenic disturbances.

| **Decade** | **1970LC-fullClim (m^3^/s)** | **Relative change to the 1970s (%)** | **2008LC-fullClim (m^3^/s)** | **Relative change to the 1970s (%)** |
| --- | --- | --- | --- | --- |
| 1970s | 7,071 | − | 9,234 | − |
| 1980s^*^ | 7,002 | −1.0 | 9,394 | +1.7 |
| 1990s | 9,047 | +27.9 | 11,839 | +28.2 |
| 2000s^+^ | 6,210 | −12.2 | 8,939 | −3.2 |

| **Decade** | **LCC-70sClim (m^3^/s)** | **Relative change to the 1970s (%)** | **LCC-00sClim (m^3^/s)** | **Relative change to the 1970s (%)** |
| --- | --- | --- | --- | --- |
| 1970s | 7,606 | − | 7,172 | − |
| 1980s^*^ | 8,342 | +9.7 | 8,584 | +19.7 |
| 1990s | 9,692 | +27.4 | 9,613 | +34.0 |
| 2000s^+^ | 10,136 | +33.3 | 9,969 | +39.0 |

* 1983 (the flood event) was excluded as an outlier and thus 1980s is the 9-year average of (1980-1989).

+ The 2008 climatology was applied for the 10^th^ year of each decade.

**Fig A1.** Decadal mean of monthly natural flow at Itaipu (data from ANA). The errorbars represent one standard deviation. The 1983 flood event is excluded.

**Fig A2.** Monthly natural flows at Itaipu Dam for 1970 to 2008 (data from ANA). The black lines in March, April, May and June indicate the positive trends that are statistically significant at a confidence level of 95% by the Mann-Kendall test. For March, the positive trend in flow is statistically significant across the complete 1970 to 2008 period, and the increasing trends in April, May and June are also statistically significant for the years before the PDO (i.e., 1970 to 1998). Note that the flood event in 1983 is excluded.

**Fig A3.** Mean monthly rainfall from Sheffield et al. (2006) meteorology. The black lines in February and March indicate the positive trends that are statistically significant at a confidence level of 95%. Year 1983 is excluded.

**Fig A4.** Climatology of ET using the 1970LC (green) and the 2008LC (red) as the initial conditions.
